# HIV persists in late coronary atheroma and is associated with increased local inflammation and disease progression

**DOI:** 10.21203/rs.3.rs-5125826/v1

**Published:** 2024-10-18

**Authors:** Laventa M. Obare, Samuel S. Bailin, Xiuqi Zhang, Kisyua Nthenge, Stephen Priest, Qi Liu, Lindsey K Stolze, Quanhu Sheng, Rama Gangula, Madelaine Behrens, Brenita Jenkins, Praveena Prasad, Kit Neikirk, Prem Prakash, Meghan Hogan, Liang Zhang, Heather K. Beasley, Jianqiang Shao, Tyne W. Miller-Fleming, Kyera V. Actkins, Mark A. Phillips, David Hubert, Jordan Malone, Cassia Labeeb, Alexander Gelbard, Antoine Chaillon, Mona Mashayekhi, Curtis L. Gabriel, Tecla Temu, Lana Olson, Angela Jones, Karen Beeri, Paxton Baker, Kenji Kawai, Saikat Kumar B. Ghosh, Laing Guo, Renu Virmani, Aloke Finn, Palak Shah, Tzushan Sharon Yang, Alexander G. Bick, Annet Kirabo, Yan R Su, Elizabeth J. Phillips, Simon Mallal, Chandravanu Dash, John R. Koethe, Sara Gianella, Melanie R. McReynolds, Tarek Absi, Antentor Hinton, Celestine N. Wanjalla

**Affiliations:** 1 Division of Infectious Diseases, Vanderbilt University Medical Center, Nashville, TN, USA; 2 Department of Biostatistics, Vanderbilt University Medical Center, Nashville, TN, USA; 3 Department of Medicine, Vanderbilt University Medical Center, Nashville, TN, USA; 4 Department of Biochemistry and Molecular Biology, The Huck Institute of the Life Sciences, Pennsylvania State University, State College, PA, USA; 5 Department of Molecular Physiology and Biophysics, Vanderbilt University, Nashville, TN, USA; 6 The Center for AIDS Health Disparities Research, Meharry Medical College, Nashville, TN, USA; 7 Department of Microbiology, Immunology, and Physiology, Meharry Medical College, Nashville, TN, USA; 8 Department of Biochemistry, Cancer Biology, Pharmacology and Neuroscience, Meharry Medical College, Nashville, TN, USA; 9 NanoString Technologies, Inc., Seattle, WA; 10 Central Microscopy Research Facility, University of Iowa, Iowa City, IA, USA; 11 Division of Genetic Medicine, Vanderbilt University Medical Center, Nashville, TN, USA; 12 Department of Integrative Biology, Oregon State University, Corvallis, OR, USA; 13 Department of Otolaryngology, Vanderbilt University Medical Center, Nashville, TN, USA; 14 Division of Infectious Diseases, University of California, San Diego, CA, USA; 15 Division of Diabetes, Endocrinology, and Metabolism, Vanderbilt University Medical Center, Nashville, TN, USA; 16 Division of Gastroenterology, Vanderbilt University Medical Center, Nashville, TN, USA; 17 Department of Pathology, Harvard Medical School, Boston, MA, USA.; 18 VANTAGE, Vanderbilt University Medical Center, Nashville, TN, USA; 19 CVPath Institute, Gaithersburg, Maryland, USA; 20 Division of Comparative Medicine, Vanderbilt University Medical Center, Nashville, TN, USA; 21 Division of Genetic Medicine, Vanderbilt University Medical Center, Nashville, TN, USA; 22 Division of Clinical Pharmacology, Vanderbilt University Medical Center, Nashville, TN, USA; 23 Department of Cardiovascular Medicine, Vanderbilt University Medical Center, Nashville, TN, USA; 24 Institute for Immunology and Infectious Diseases, Murdoch University, WA, Western Australia; 25 Department of Pathology, Microbiology and Immunology, Vanderbilt University Medical Center, Nashville, TN; 26 Department of Biomedical Informatics, Vanderbilt University Medical Center, Nashville, TN; 27 Department of Cardiac Surgery, Vanderbilt University Medical Center, Nashville, TN, USA

**Keywords:** Atherosclerosis, cardiovascular disease, HIV, CD163, CXCL12, ABCA1

## Abstract

Chronic inflammation contributes to the prevalence of cardiovascular disease in people living with HIV (PLWH). The immune mechanisms driving atherosclerosis progression in PLWH remain unclear. This study conducted comprehensive assessments of medium-sized coronary arteries and aorta from deceased PLWH and controls without HIV using DNA/RNA assays, spatial transcriptomics, and high-resolution mass spectrometry. Findings revealed more significant inflammation correlated with higher HIV copy numbers in late atheroma of PLWH. Enhanced *CXCL12* and decreased *ABCA1/ABCG1* expression in CD163^+^ macrophages were co-localized in coronaries of PLWH, suggesting a reduction in plasma lipoprotein clearance compared to controls. Spatial analyses identified potential therapeutic targets by revealing inflammatory changes in medium-sized arteries and the aorta. We examined the relationship between atherosclerotic phenotypes and inflammatory gene expression in Vanderbilts Biobank to study these findings in a larger clinical cohort. This established a significant association between *ABCA1* and *CXCL12* gene expressions with atherosclerosis, partly influenced by HIV.

## Introduction

People living with the human immune deficiency virus (PLWH) have a higher risk of developing cardiovascular disease (CVD) compared to people without HIV (PWoH), which is not fully explained by differences in traditional risk factors. This risk persists despite viral suppression with antiretroviral therapy (ART) as measured by plasma viral load.^1–5^ PLWH on ART display higher levels of inflammation compared to PWoH.^6,7^ Studies have shown that ‘elite controllers’ (i.e., PLWH who maintain low or undetectable plasma viremia in the absence of ART) exhibit higher carotid intimal media thickness compared to PWoH after adjusting for traditional risk factors, suggesting that ART is not likely the primary driver of vascular disease.^8^ In PLWH, 18F-fluorodeoxyglucose positron emission tomography (FDG-PET) imaging demonstrated that aortic and carotid artery inflammation was higher when compared to PWoH.^9,10^ Greater tracer uptake on FDG-PET has been associated with higher hs-CRP, IL-6, blood CX3CR1^+^ monocytes, and potentially CX3CR1^+^ CD4^+^ T cells.^9,10^

Chronic inflammation due to cytomegalovirus (CMV) has also been linked to accelerated atherosclerosis.^11,12^ Most PLWH are co-infected with CMV and have inflated CD4^+^ and CD8^+^ T cells that control CMV replication.^11,12^ A higher percentage of CMV-specific CD8^+^ T cells in PLWH has been associated with increased carotid intima-media thickness. Higher anti-CMV IgG titers were also associated with subclinical carotid artery disease and increased mortality from coronary heart disease.^13^

Among PLWH, lower persistent CD4^+^ T cell counts have been linked with higher rates of non-AIDS diseases, including CVD.^14^ CD4^+^ T cell counts of less than 200 cells/mm^3^ have also been associated with greater arterial stiffness^15^ and carotid plaque.^16^ However, endothelial function normalizes when CD45RA^+^ CD4^+^ cells (likely CMV-specific TEMRA) are entirely lost.^17^ Notably, higher absolute CD4^+^ T cell counts in PLWH have also been associated with cardiovascular aging in PLWH.^18,19^ A potential explanation for this paradox may be the expansion of a subset of cytotoxic virus-specific T cells in the presence of ART, which are highly inflammatory and atherogenic.

Although inflammation plays an essential role in the development of atheroma, few studies have evaluated arterial plaques in persons with chronic viral infections such as HIV. We employed a multimodal approach to deeply phenotype medium-sized coronary arteries and aortas using immunohistochemistry, spatial transcriptomic profiling technologies, and tissue metabolomics and lipidomic analyses. We also leveraged a large DNA biorepository (BIOVU) to identify genetic variants in inflammatory genes associated with CVD risk in PLWH and study cardiovascular disease mechanisms.

## RESULTS

### Characterization of coronary artery morphology from PLWH and PWoH

We analyzed medium-sized coronary artery FFPE blocks from 13 deceased PLWH (median age 52.5 years) and 15 deceased PWoH (median age 51 years) (**Table S1**). Participants were matched for age, sex, and plaque characteristics. Approximately 50% of each group were females. There were more Caucasians among donors without HIV (9/15) compared to PLWH (4/13). Plaque grading included pathologic intimal thickening (PIT) and early and late atheroma (**Table S1**). Cardiac death categories, as applicable, are detailed in **Table S1**. Plaque morphology, including plaque area and stenosis, did not differ between individuals with and without HIV **(Figure S1A Figure S1B).** Plaque area correlated with stenosis, irrespective of HIV status, and did not correlate with age **(Figure S1C, D)**. The relationship between plaque area and stenosis varied by sex, showing a stronger correlation in females **(Figure S1E, F)**.

### HIV is present in atheroma and persists in higher copy numbers with disease progression

**Figure 1.**
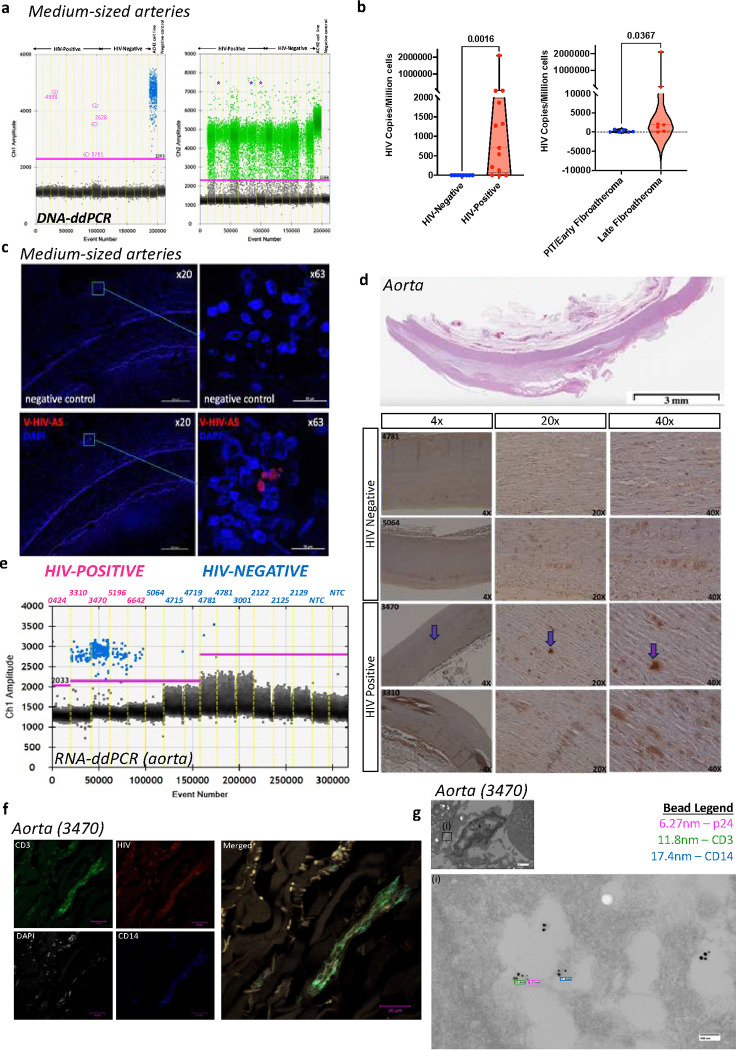
DNA/RNA and protein assays localize HIV within the coronary arteries and the aorta of PLWH **a**, Fluorescence intensities depict DNA droplets ddPCR analysis of medium-sized coronary arteries of seven donors with HIV and six without HIV. The blue droplets on the left are positive for HIV LTR DNA, fluorescence intensity versus droplet number in each droplet. This was multiplexed with RNaseP as the housekeeping gene depicted on the right (green). The positive droplet threshold was determined using the no template controls on the same run. **b**, Violin plots depict differences in HIV viral copies by sample group (left) and PLWH alone, comparing pathologic intimal thickening (PIT)/early to late atheroma. **c**, Image of HIV detected in coronary arteries using RNAscope probe HIV-Gagpol. **d**, We stained aorta samples for Immunohistochemistry analysis with anti-HIV p24. **e**, Fluorescence intensities depict droplets positive for HIV RNA (aorta samples from five PLWH and nine PWoH deceased donors) as measured by qPCR. **f**, Image of HIV detected in coronary arteries using RNAscope probe HIV-Gagpol. **g**, Confocal microscopy of the aorta, using an antibody against HIV p24, anti-CD14, anti-CD3, and DAPI. **h**, Transmission electron microscopy of FFPE blocks combined with immunogold labeling of the 3470 sample shows colocalization of HIV p24 (6nm), CD3 (12nm), and CD14 (18nm). Statistical analysis was done using the Mann-Whitney U test.

We quantified HIV copies per million cells (immune and nonimmune) in 10/13 coronary artery samples from PLWH using DNA-based droplet digital PCR (ddPCR). We did not have access to information on virological suppression from the donors of the coronary arteries (Figure 1a). When stratified by plaque type, late atheroma contained significantly more copies of HIV compared to early atheroma (Figure 1b). We examined six coronary arteries using RNA in situ hybridization (RNAscope) and specific probes for the HIV-1 *Gag* gene. We found evidence of HIV in the adventitia in one out of six tissue samples (Figure 1c). Notably, in a separate procedure using DNA ddPCR, HIV was detected in four of the six coronary arteries previously tested by RNAscope.

We further phenotyped aortas from a subset of the deceased PLWH (n=five, one female, age 36–72 years of age) and deceased PWoH (n=nine, one female, age 24–69 years) (**Table S2)**. HIV-p24 protein was detected in the aortas of PLWH, although high tissue necrosis reduced IHC sensitivity and specificity (Figure 1d). We detected HIV RNA in 4/5 PLWH aortas using ddPCR (Figure 1e), and confirmed HIV RNA presence in all five aorta’s with RNAscope (Figure 1f). Regarding localization, HIV was present in the same area as CD14^+^ and CD3^+^ cells as visualized by confocal microscopy (Figure 1g, top). Additionally, immuno-transmission electron microscopy (iTEM) imaging of serial blocks from the same participant who had detectable HIV (based on RNAscope) provided further confirmation (Figure 1h, bottom). One of the five PLWH from whom we obtained the aorta samples had confirmed viral suppression in their death report (viral load <40, with CD4^+^ T cell count of 680). The presence of latent and replicating HIV in medium and large-sized coronary arteries, even among a participant on ART with viral suppression, suggests that HIV may drive persistent inflammation within coronary arteries. ^20^

### HIV in coronary arteries is associated with local inflammation

Using IHC, we stained the coronary arteries with antibodies to quantify T cells (CD3/CD4/CD8), monocytes/macrophages (CD68/CD163), and adhesion molecules (vascular cell adhesion molecule 1 VCAM-1). Additional markers included granzyme B (GZB), stimulator of interferon-gamma (STING), and markers associated with cell trafficking to inflamed endothelium (CX3CR1). Representative CD3, CD4, and CD68 staining images are shown (**Figure S2a-b**). First, we compared differences in immune cells by atheroma stage and observed that earlier stages had significantly higher proportions of CD3^+^ and GZMB^+^ cells than late atheroma (**Table S3**). CD163 expression, on the other hand, was higher in late atheroma (**Table S3**). Plaque area and % stenosis were also higher in late atheroma compared to early atheroma.

We compared the proportions of immune cells in the coronary arteries by HIV status. Coronary arteries from PLWH had a higher percentage of CD68^+^ cells (1.58% [0.28, 2.94]) compared to PWoH (median 0.33% of total plaque area [0.13, 2.11]) though this was not statistically significant (p=0.05). CD3^+^ T cell proportions did not differ by HIV status (0.04% [0.03, 0.13] in PLWH vs. 0.19% [0.11, 0.34] in PWoH, p=0.39). Similarly, CD4^+^ (0.6% [0.02, 1.54] in PLWH vs. 0.31% [0.1, 0.51] in PWoH, p=0.09) and CD8^+^ T cells (0.02% [0.003, 0.24] in PLWH vs. 0.17% [0.08, 0.28] in PWoH, p=0.18) did not differ significantly by HIV status. Although there was a trend towards more immune cells in plaques from PLWH, our study was underpowered due to the small sample size of coronary arteries (**Figure S2c-d**).

**Figure 2.**
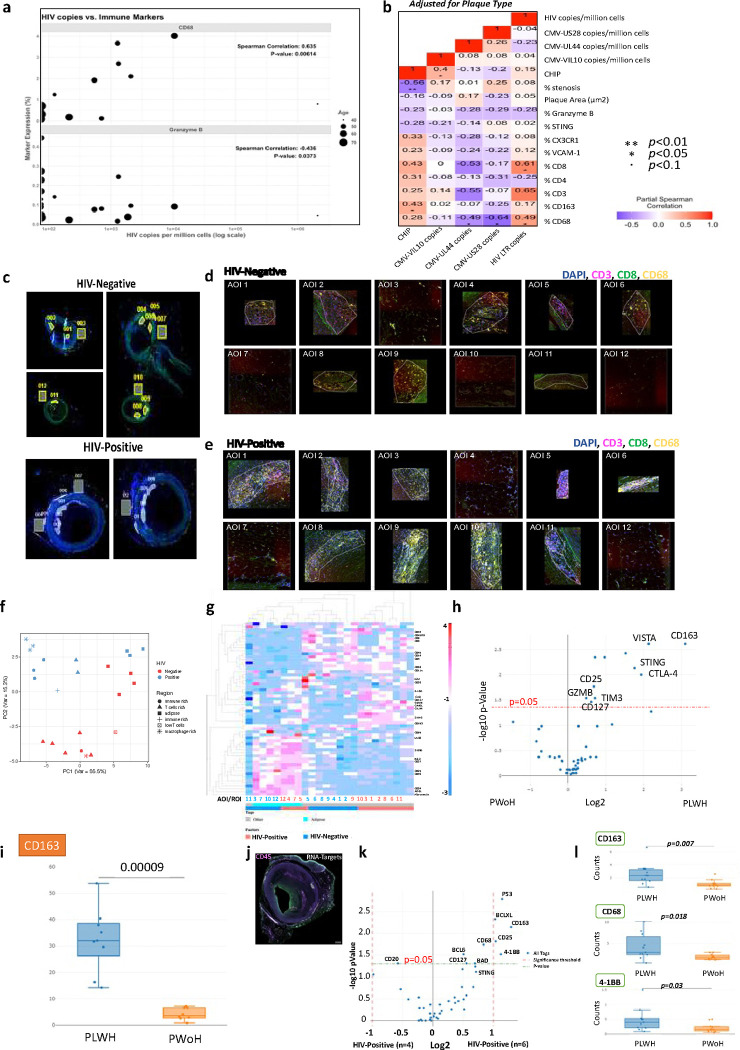
Inflammation within coronary arteries of PLWH is associated with the HIV copy number. **a,** Bubble plot shows a correlation between HIV copies and immune markers, with the size of the bubble depicting the age of the donors (n=28). **b**, The heat map shows an adjusted correlation between immune markers with clonal hematopoiesis of indeterminate potential (CHIP), CMV DNA copies, and HIV DNA copies adjusted for plaque type (right). **c**, Fluorescent images of coronary arteries from PLWH and PWoH matched at the atheroma stage (Early atheroma) using in GeoMX analysis. Areas of interest (AOI 1–12) were selected by drawing geometric regions within the plaque in the advenqqa and perivascular adipose qssue. The coronary arteries were stained with anq-CD3 (magenta), anq-CD8 (green), and anq-CD68 (yellow). SYTO 83 nuclear staining was included to visualize all cells (blue). **d-e**, The twelve areas selected per sample are shown by HIV status. **f**, PCA plot shows the distribuqon of AOIs by region and HIV status. **g**, A heatmap groups AOI segments with similar protein expression. **h**, Box plots show differences in the expression of select proteins in the different AOIs by HIV status. **i**, The volcano plot shows differenqal *protein* expression of all AOIs by HIV status. We used the GeoMX immune pathways RNA panel (84 targets) to compare coronary arteries from PLWH (n=6) to PWoH (n=4). For this analysis, coronary arteries were stained using a custom panel with anti-CD45 (magenta) and PanCK (Green). **j**, A similar transcriptomic analysis was performed on ten coronary arteries with AOIs selected based on CD45 expressions (PLWH n=six, PWoH n=four). **k**, Differenqal expression of *RNA targets* is shown by the volcano plot. **l**, Box plots show the contribuqon of AOIs for select genes *– CD163, CD68*, and *4–1BB*: statistical analysis, Wilcoxon test, Spearman rank, and Partial Spearman rank analysis. Significance was determined by Mann Whitney test with BH correcqon, * p < 0.05, ** p < 0.01, * *** p < 0.001, **** p < 0.0001.

To establish the link between HIV presence in coronary arteries and inflammation, we observed that HIV copies in coronary arteries were positively correlated with CD68 expression and negatively with Granzyme B (Figure 2a). This relationship between HIV copies and CD68 expression remained significant after adjusting for age (**Figure S3a**) and plaque type (Figure 2b, **Figure S3b**). Additionally, detectable HIV in coronary arteries was associated with CD8 expression when adjusted for plaque type.

Clonal hematopoiesis of indeterminate potential (CHIP) is associated with CVD and is more prevalent in PLWH.^21^ We detected CHIP in 46% of the donors. Among those with CHIP, 60% were PLWH, and 40% were PWoH (**Table S4**). The top differentially expressed genes in PLWH were *TP53, ASXL1, TET2*, and *DNMT3A*, and the top genes in PWoH were *ASXL1* and *TET2*. We included CHIP as a binary variable and found that coronary arteries with CHIP were associated with a higher proportion of CD163 when adjusted for plaque type (Figure 2b). This relationship was decreased when adjusted for age (**Figure S2a-b**). CHIP was negatively associated with % stenosis even after adjustment of age (**Figure S2a**) and plaque type (PIT, early and late atheroma) (Figure 2b). We did not look at differences in CHIP by HIV status due to a limited number of samples.

Given the proposed role of CMV infection in aging and CVD among PLWH, we also measured the association between CMV viral gene copies and inflammatory markers expressed in coronary arteries. We did not detect many copies of CMV in the coronary arteries, and there were no significant differences in the CMV copy numbers from PLWH or PWoH (**Figure S4a-c**). Furthermore, there was no correlation between HIV copies and total CMV copies in coronary arteries (**Figure S4d**). CMV copy numbers quantified using UL44 and US28 were negatively associated with % CD68 expression in all samples (Figure 2b). CMV viral IL-10 copies were associated with CHIP.

Previous studies have shown that CD8^+^ T cells and macrophages are essential in both early and advanced stages of atherosclerosis.^22^ We hypothesized that HIV-related differences, including immune senescence, are amplified with HIV and contribute to increased plaque formation and progression with HIV. We leveraged spatial transcriptomics analysis and a larger panel of immune protein markers (GeoMx^®^ Immuno-Oncology Protein Panels, n=96 different proteins) to assess the spatial relationships between immune and non-immune cells in coronary arteries. Coronary plaque sections from two donors (one PLWH and one PWoH) were matched by age, atheroma stage, sex, and percent of immune cells, as seen by IHC. After staining with fluorescently tagged antibodies (CD3, CD8, CD68, and DAPI), sequential areas of interest (AOI) were selected (Figure 2c, **Figure S5**). There was significant heterogeneity in areas within the plaque as some regions had predominantly macrophages, and others had predominantly CD3^+^/CD8^+^ cells (Figure 2d–e). We selected AOIs (within the intima, media, adventitia, and perivascular adipose tissue) matched by CD3, CD8, and CD68 protein expression as measured by immunofluorescence (**Figure S6**).

HIV status did not clearly distinguish all the AOIs selected in the samples, as demonstrated in the PC plot (Figure 2f). Adipose tissue AOIs were similar, while other regions were separated by HIV status. Differential protein expression of all AOIs by HIV status showed higher *STING, CD163*, V-domain immunoglobulin suppressor of T cell activation (*VISTA*), and cytotoxic T-lymphocyte-associated protein 4 (*CTLA-4*) (*p* < *0.05*) in coronary plaques from PLWH (Figure 2g–h). The most highly expressed proteins in the AOIs from PLWH included *CD163* and *VISTA* (Figure 2i). A similar analysis comparing coronary arteries from PLWH and PWoH using RNA gene probes against immune markers in AOIs selected by CD45 expression (Figure 2j) revealed that several genes were significantly higher in coronary arteries from PLWH, including *CD163*, *41BB*, *BCLXL*, and *TP53* (Figure 2k). Box plots show differences in the RNA transcript levels of *CD163, 41BB*, and *CD68* (Figure 2l). The spatial depiction of *CD163* shows the expression in different ROIs and HIV status (**Figure S7a-b**). Most selected AOIs with high *CD163* gene expression were within the adventitia. CD163 is a scavenger receptor of hemoglobin/haptoglobin complexes expressed on macrophages, and the soluble form is higher in the peripheral blood of PLWH than in PWoH.^23^ Consistent with previously published work showing a relationship between soluble CD163 and CVD in PLWH, we observed higher RNA and protein levels of CD163 in medium-sized coronary arteries from PLWH compared to PWoH.

### Spatial transcriptomics in the aorta from PLWH also shows increased CD163 and colocalization with HIV RNA expression

**Figure 3.**
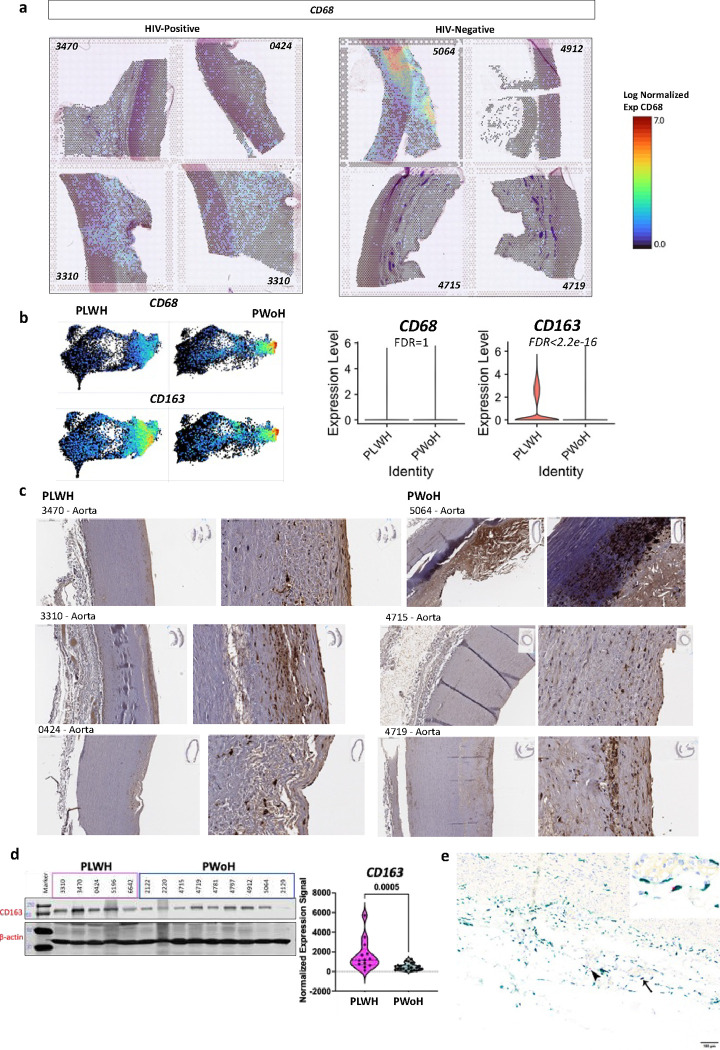
CD163 is higher in the aorta from PLWH compared to PWoH and colocalizes with HIV RNA **a**, Thoracic aorta obtained from deceased donors were stained with H&E stains and processed for Visium spatial gene expression analysis. The log normalized expression of *CD68* is shown with a spatial resolution. **b**, The combined HIV-positive and HIV-negative aorta are projected to a UMAP representing each spot that captures data (55μm per spot). Both *CD68* and *CD163* expression as shown on the UMAPs. Violin plots show the log-normalized expression of *CD68* and *CD163* in all eight samples by HIV status. **c**, IHC, and **d**, western blot showing *CD163* protein expression. Violin plots show *CD163* expression normalized to the housekeeping proteins β-actin from 3 separate runs. **e**, Dual detection of CD163 protein expression by immunohistochemistry (green) and HIV RNA hybridization (red) within the aortic adventitia. HIV signals were detected adjacent to (arrowhead) or within CD163-labeled macrophages (thin arrow, inset).

We analyzed the aorta from three deceased donors with HIV and four without HIV (**Table S2**). *CD68* gene expression by HIV status is shown for individual aorta samples (Figure 3a) and combined by HIV group status projected onto a UMAP (Figure 3b). *CD68* was similar between the two groups. However, *CD163* expression was significantly higher in the aorta of PLWH. Analysis by immunohistochemistry shows the distribution of *CD163* gene expression in the aorta obtained from PLWH and PWoH (Figure 3c). There is a significant heterogeneity in the expression of CD163, with expression more concentrated along the adventitial layer, in areas with pathology and near the endothelial cell lining of the intima. We extracted protein from flash-frozen aorta samples and quantified *CD163* protein expression (Figure 3d). Despite heterogeneity in the expression of *CD163* depending on the tissue sampled, repeated western blot analysis (four times) consistently showed higher *CD163* expression in the aorta from PLWH (Figure 3d, **violin plot**). We detected HIV RNA expression colocalized with *CD163*-expressing cells (Figure 3e). Another marker of interest was *CD209*, a C-type lectin receptor expressed on macrophages thought to be a receptor for HIV transmission.^24^ While CD209 protein levels were not significantly higher in PLWH compared to PWoH, we observed a positive correlation in *CD163* and *CD209* expression (**Figure S8a-d**).

### Enhanced CXCL12 and decreased ABCA1/ABCG1 expression in CD163^+^ of PLWH in a spatially restricted manner

**Figure 4.**
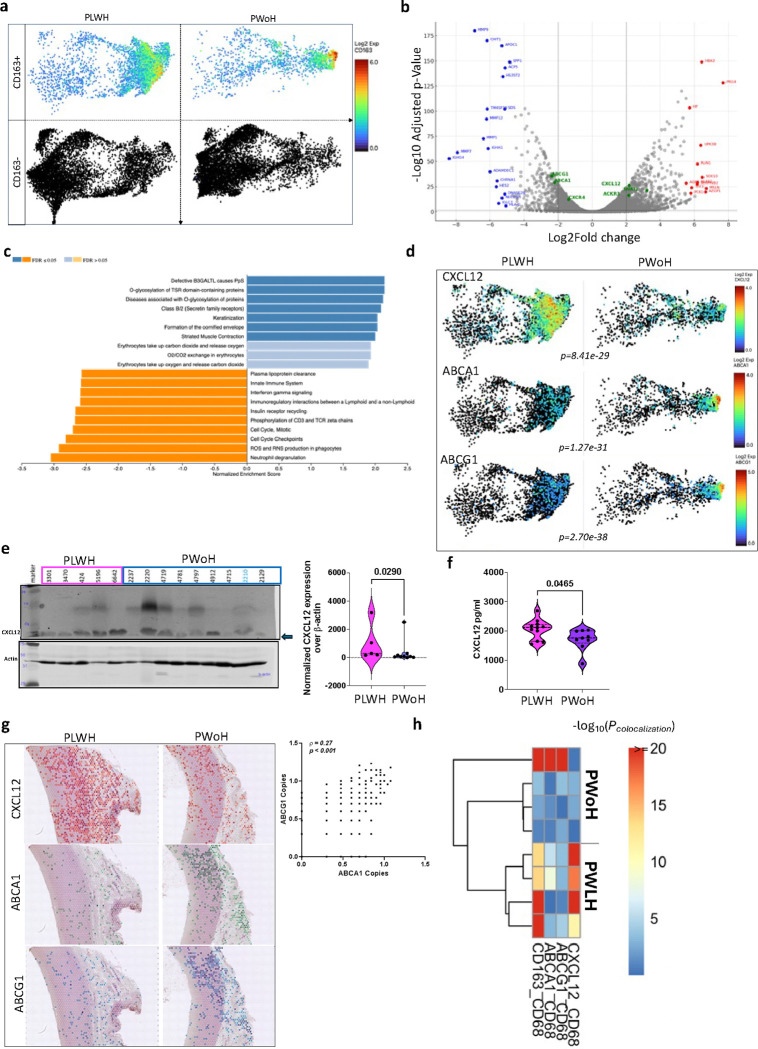
Reduced plasma lipoprotein clearance in the aorta of PLWH **a,** We compared regions with CD163^+^ expression to regions without CD163^+^ expression in the aorta of PLWH (n=4) and PWoH (n=4). **b**, Differential gene expression was performed using a negative binomial test (edgeR), highlighting genes higher in CD163^+^ macrophages from PLWH in red and lower genes in blue. **c**, GSEA analysis with the Reactome pathways shows several pathways different by HIV (blue are upregulated and orange are downregulated pathways in CD163^+^ macrophages from PLWH). **d**, UMAPs show the RNA transcript levels of several ABC transporters, including CXCL12, ABCA1, and ABCG1 in the aorta. **e**, Western blot was used to quantify CXCL12 protein expression in the aorta from PLWH and PWoH, depicted in the violin plot. **f**, Plasma CXCL12 was measured in the plasma of diabetic PLWH and PWoH. **g**, Representative images show the gene expression of *CXCL12, ABCA1,* and *ABCG1* in the same regions of the aorta of PLWH and PWoH. ABCA1 is correlated to *ABCG1* gene expression (Spearman rank). **h**, Heatmap shows the spatial colocalization of *CXCL12, ABCA1, ABCG1*, and *CD163* in CD68^+^ macrophages quantified by SpaGene, a scalable and model-free method.^32^ The color scale indicates the significance of colocalization (blue: low significance, red: high significance).

CD163^+^ macrophages have been associated with plaque progression due to high levels of HIF1a expression, leading to increased angiogenesis and plaque vulnerability.^25^ Furthermore, CD163^+^ macrophages can induce endothelial-to-mesenchymal transition within the fibrous cap^26^ and restrain vascular calcification, as demonstrated in CD163^−/−^ ApoE^−/−^ mice and in vitro co-culture experiments.^27^ Given the increase in CD163^+^ macrophages, some infected with HIV, we analyzed and compared *CD163*^+^ cells in the aorta of PLWH and PWoH (Figure 4a). Several genes were differentially expressed (Figure 4b), which enriched for several pathways, including O-glycosylation of proteins and significantly lower lipoprotein clearance in the aorta from PLWH (Figure 4c).

We hypothesized that dysregulated lipoprotein clearance mediated by CD163^+^ macrophages might be necessary for atherosclerosis progression among PLWH. CXCL12 is thought to promote atherosclerosis progression by signaling downstream of CXCR4. ^28^ CXCL12 has also been shown to interact with the transcription factor TCF21 to inhibit ABCA1 expression.^29^ On the other hand, ATP-binding cassette transporter A1 (ABCA1) and ATP-binding cassette transporter G1 (ABCG1) are lipid transporters that modulate lipid homeostasis and, when dysregulated, lead to dyslipidemia.^29^ We observed higher *CXCL12* transcripts in the aorta of PLWH and lower *ABCA1* and *ABCG1* compared to the PWoH control (Figure 4d). CXCL12 protein quantification by Western blot was also higher in the aorta of PLWH than in PWoH (Figure 4e). We leveraged an existing well-characterized cohort of PLWH and PWoH and measured CXCL12 in the plasma.^30,31^ CXCL12 levels were higher in the plasma of diabetic PLWH than diabetic PWoH (Figure 4f). The spatial expression of *CXCL12, ABCA1,* and *ABCG1* are shown in representative images from PLWH and PWoH (Figure 4g). There was a positive correlation between *ABCA1* and *ABCG1* expression in the aorta irrespective of HIV status. We analyzed the spatial colocalization of *CXCL12, CD163, ABCA1,* and *ABCG1* with CD68^+^ macrophages using SpaGene.^32^ The four samples from PLWH, *CD163*, and *CXCL12* had significantly greater colocalization with CD68+ macrophages compared to ABCA1 and *ABCG1*. Conversely, in only one sample from PWoH was there significant colocalization between CD163 and CD68+ macrophages, and its colocalization pattern differed from that of the PLWH samples. In this case, *ABCA1* and *ABCG1*, rather than *CXCL12*, showed significant colocalization with CD68+ macrophages (Figure 4h).

### CellChat predicts the CXCL12 signaling pathway between fibroblasts and TCF7^+^ monocytes and lymphoid cells within coronary arteries

**Figure 5.**
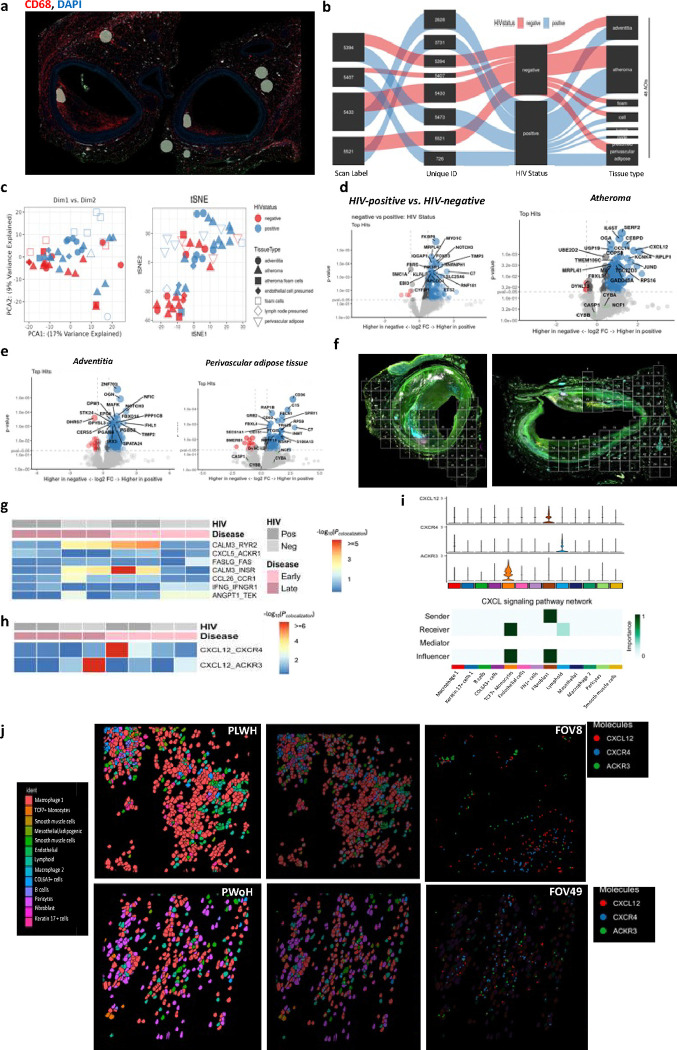
CXCL12 expression within coronaries is most detectable in atheroma and is predicted to interact with CXCR4 and ACKR3 **a,** Regions of interest in coronary arteries were selected based on CD68 expression for spatial analysis using the GeoMX platform, as shown by the representative image. **b**, The Sankey plot shows the ROI distribution across different coronaries sections from four PLWH and four PWoH. The width of the lines is proportional to the representation of the different regions. **c**, Nanostring GeoMX whole transcriptomic sequences were obtained from selected regions of interest. PCA and tSNE plots show regions of interest that co-cluster together based on similarities in gene transcripts. **d**, The volcano plots show differential gene expression by HIV status in all regions and atheroma, **e**, adventitia, and perivascular adipose tissue. **f**, Two representative images showing the fields of view of regions selected in the coronary arteries from PLWH and PWoH. **g**, Heatmap showing the spatial colocalization of genes in coronary arteries, stratified by HIV status (Pos: HIV-positive, Neg: HIV-negative) and atheroma stages (Early vs. Late). The color scale indicates the significance of colocalization (blue: low significance, red: high significance). **h**, Heatmap showing colocalized expression of *CXCL12* and its receptors CXCR4 and ACKR3 across HIV groups and atheroma stages. **i**, The violin plots show the gene counts of *CXCL12, CXCR4*, and *ACKR3* in each cluster on the x-axis by color code. The heatmap right below shows the predicted CellChat ligand-receptor interactions. **j**, Representative CosMx fov’s (8 and 49) from coronary arteries of PLWH and PWoH with all cells’ x and y locations. Individual transcripts (*CXCL12* - red, *ACKR3*-green, and CXCR4-blue) are shown on the fov images depicted as molecules. The figure legends are color-coded to depict the different cell types and molecules.

CD68 staining of the coronary arteries (Figure 5a) was used to select matched AOIs (Figure 5b). When visualized in two dimensions using PCA, there was no distinct separation by HIV status within the first two principal components, suggesting that in the selected regions, HIV did not have a strong, linear, isolated impact on the variance of the selected AOIs. Each AOI was distributed across the principal components, which may reflect each region’s unique gene expression patterns or specific characteristics (Figure 5c). The t-SNE plot, on the other hand, also indicated no clear separation based on HIV status. However, there seemed to be a subtle grouping suggesting similar high-dimensional characteristics by HIV status. Regardless of region, differential gene expression by HIV status revealed statistically significant differences (Figure 5d). *CXCL12, IL6ST, CCL14, CEBPD, and KCNK4* were more expressed in the atheroma of coronaries from PLWH (Figure 5d). While several genes including *CDK11A, CCND2, TARDBP*, *GMPS, G0S2, DPYD, RBM8A, NIPAL2,* and *APMAP were higher in PWoH* (Figure 5d–e).

We analyzed gene expression within coronary arteries at a single-cell level with spatial resolution using CosMx SMI. We selected four PLWH and four from PWoH, including early and late atheroma. Several fields of view (fov) were chosen in each coronary artery to cover the perivascular adipose, media, and intima (Figure 5f). We used machine learning segmentation to resolve the spatial data from the four coronary arteries at a single-cell resolution. We used SpaGene to compare ligand-receptor interactions within the coronary arteries of PLWH and PWoH at early and late atheroma stages (Figure 5g–h). Interactions associated with immune regulation, inflammation, and vascular remodeling—including CALM3_RYR2 (calcium signaling), CXCL5_ACKR1 (immune cell migration), FASLG_FAS (apoptosis), and ANGPT1_TEK (angiogenesis)—exhibited different interaction strengths between PLWH and PWoH at either early or late atheroma stages (Fig. 5g). Additionally, the CXCL12-CXCR4 interaction was low in both PLWH and PWoH during late-stage atheroma. In contrast, the interaction was notably stronger in PLWH at early-stage atheroma. In comparison, CXCL12-ACKR3 interaction was stronger in coronaries from PWoH at late-stage atheroma (Fig. 5h). These findings suggest that HIV exerts a distinct impact on ligand-receptor interactions at early and late stages of atheroma. Using CellChat, fibroblasts within the coronary arteries were predicted to be the primary senders of the CXCL12 signal. TCF7^+^ cells identified as possible monocytes due to co-expression of CD14 (**Figure S9**) were the most significant recipient cells through the ACKR3 receptor, followed by lymphoid cells through CXCR4 (Figure 5i). Spatial analysis shows that CXCR4 expression is predominantly in niche seven (green), in plaque (**Figure S10**). *CXCL12, ACKR3*, and CXCR4 RNA transcripts are shown in representative fov (Figure 5j), demonstrating the expression of the colocalization of the gene transcripts. Other highly expressed genes within the atheroma are also demonstrated at the molecular level (**Figure S11**). In summary, we show the differential expression of *CXCL12* in medium-sized coronary arteries and its receptors with spatial resolution. This suggests a role for the CXCL12-CXCR4-ACKR3 axis in the immune microenvironment and atherosclerosis in PLWH.

### Low retinoic acid and high triglycerides are potential inflammation drivers within large-sized arteries.

**Figure 6.**
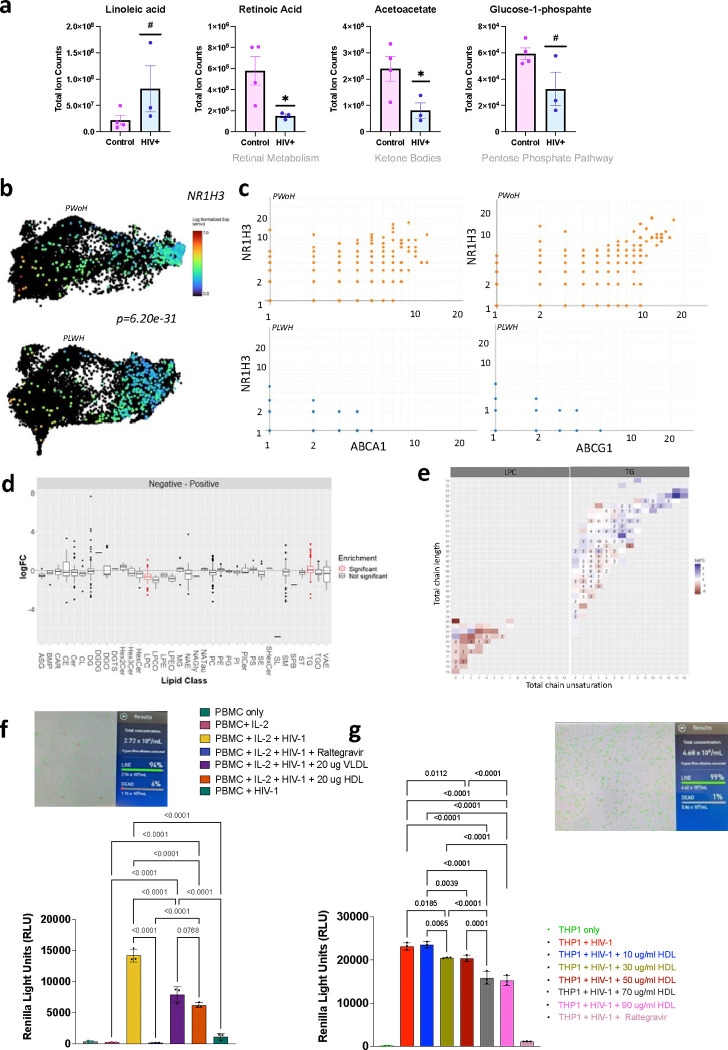
Reduced retinoic acid and increased triglycerides in the Aorta of PLWH compared to PWoH. **a,** Bar plots show select metabolites compared by HIV status (* p < 0.05, and *# p < 0.1 with n=4 PWoH as controls and n=3 PLWH*)**. b,** UMAP shows the expression of the *NR1H3* gene by HIV status in CD163^+^ cells. **c,** Correlation plots show the relationship between *NR1H3, ABCA1*, and *ABCG1* in CD163^+^ cells in the aorta of PWoH and PLWH. **d.** The box plot shows the log2-fold change in lipid class enrichment in the aorta of PLWH, with PWoH as the reference. **e,** Heat maps show enriched lipid classes based on HIV status. **f,** The bar plot shows PBMCs infected with pseudo-HIV expressing VSV glycoprotein and Renilla luciferase and incubated with antiviral therapy (Raltegravir, Ral), 20μg/ml VLDL, and 20μg/ml HDL. The picture shows the viability of PBMCs infected with HIV and incubated with lipids (VLDL or HDL). **g**, The bar plot shows THP1 monocytes infected with pseudo-HIV expressing VSV glycoprotein and Renilla luciferase and incubated with antiviral therapy (Raltegravir, Ral), 20μg/ml LDL, 20μg/ml VLDL, and 20μg/ml HDL. The picture shows the viability of the cells. Statistical analysis in c, Spearman rank correlation analysis, f,g. Ordinary one-way ANOVA with Tukeys multiple comparison.

Low retinoic acid has been associated with an increased risk of coronary events among middle-aged men.^33^ Vitamin A supplementation in ApoE^−/−^ mice was shown to upregulate ABCA1 and ABCG1 lipid transporters via the liver X receptor-α (LXRα, NR1H3), slowing down atherosclerotic plaque progression.^34^ We observed that the aorta from PLWH had lower levels of retinoic acid (retinol metabolism) and acetoacetate (ketone bodies) than the aorta from PWoH (Figure 6a). The latter may indicate less fatty acid oxidation in large vessels of PLWH, which agrees with PET imaging studies showing greater radiolabeled glucose uptake in the aorta of PLWH.^35^ Linoleic acid was higher in the aorta of PLWH but not significant. The *NR1H3* transcript was higher in the CD163^+^ cells in the aorta of PWoH (Figure 6b) than PLWH and correlated with ABCA1 and ABCG1 expression in PWoH (Figure 6c). Among lipids, triglycerides (TG) were higher in the aorta of PLWH, and lysophosphatidylcholines (LPC) were higher in the aorta of PWoH (Figure 6d). There was no difference in lipid chain by HIV status (Figure 6e).

Viable PBMCs from PWoH were infected with increasing copies of pseudo-HIV (labeled VsVG Luc) in the presence of IL-2 with an increase in the viral load, which was inhibited at the highest multiplicity of infection by the addition of raltegravir (RAL). Adding VLDL and HDL to PBMCs lowered the infection (Figure 6f). THP1 monocytes were treated similarly, adding LDL, VLDL, and HDL. We observed decreased HIV infection with HDL in a dose-dependent manner, which was not due to reduced viability in the THP1 cells. Taken together, the metabolomic analysis of the aorta suggests that vitamin deficiencies may amplify inflammatory changes. Vitamin A/retinoic acid has a significant role in regulating the innate and adaptive immune system.^36^ The finding of lower retinoic acid in the aorta from PLWH goes along with studies showing lower vitamin A levels in asymptomatic PLWH compared to PWoH, which does not appear to be related to lower intake.^37^ While HIV may alter lipid accumulation, as previously described^38,39^, we did not see an increase in HIV replication by adding LDL, VLDL, or HDL to PBMCs and THP1 cells. We did not test whether this promotes latency, which is another possibility.

### Genetic variations in CXCL12 may contribute to gene expression of inflammatory genes associated with atherosclerotic phenotypes and are in part driven by PLWH

To examine the relationship between atherosclerotic phenotypes and inflammatory gene expressions in a clinical population, we leveraged the genetic and phenotype information available in the BioVU biobank. Across 77,678 individuals, we calculated genetically regulated gene expression (GREX) for *ABCA1, CD163*, and *CXCL12* based on genotype data and gene expression models derived from the GTEx dataset (see Methods, **Table S6**). Within the biobank population, we stratified our data by genetic ancestry into individuals of European and African ancestry. GREX of *ABCA1* and *CXCL12* was significantly associated with atherosclerotic phenotypes, including ischemic heart disease and coronary atherosclerosis in individuals of European genetic ancestry (n=65,364 , p<0.0044, **Table S7**). We also found significant associations between the GREX of *CXCL12* and coronary atherosclerosis in individuals of African genetic ancestry (n=12,314, p=0.0035, **Table S8**). Additionally, we found nominal associations between *ABCA1*, *CD163*, and *CXCL12* GREX with ischemic heart disease, coronary atherosclerosis, and myocardial infarction in individuals of European ancestry and nominal associations between *ABCA1* and *CXCL12* GREX with ischemic heart disease, coronary atherosclerosis, and myocardial infarction in individuals of African ancestry (p < 0.05, **Table S7, Table S8**).

Based on our findings that the GREX of *CXCL12* is significantly associated with atherosclerotic phenotypes within the BioVU population by linear regression models, we next performed a sensitivity analysis to test whether this association is being driven by BioVU individuals with an HIV diagnosis. We adjusted our regression model to include a covariate for HIV diagnosis extracted from the electronic health records using ICD9/10 billing codes (see Methods). After adjusting our model for HIV diagnosis, we find that the association p-value drops one order of magnitude and no longer meets the Bonferroni-corrected threshold in both individuals of European and African ancestry (p = 0.02801, p= 0.02799, respectively, **Table S9**); however, these associations are still nominally significant. Overall, these findings suggest that genetic variations that contribute to gene expression of inflammatory genes are associated with clinical diagnosis of atherosclerotic phenotypes. These associations are, in part, driven by individuals with HIV. Further research is needed to validate these findings and understand their biological and clinical significance.

## DISCUSSION

The present study used a multimodal approach to investigate medium-sized coronary arteries and aorta from deceased individuals with and without HIV. To understand mechanisms that promote plaque inflammation and atherosclerosis among PLWH, we conducted a phenotypic analysis of medium-sized coronary arteries and the aorta and examined metabolites and lipids. Our key findings support the role of an increased inflammatory response in the coronary arteries and aorta of PLWH, which is likely partly associated with the presence of HIV in immune cells recruited to the tissues. There was significant heterogeneity in immune cell composition and distribution within the plaque. Some regions had predominantly macrophages, and others had a combination of CD3^+^/CD8^+^ cells. This heterogeneity was similar in both arteries from both PLWH and PWoH, suggesting that immune cell heterogeneity is a common feature of atherosclerotic plaques. While different cell types are present, we found many monocyte/myeloid cells among smooth muscle cells and fibroblasts. *CD163*+ macrophages were more prominent in the coronary arteries and aorta of PLWH than PWoH and could help maintain the HIV reservoir within the arteries. Notably, *CD163*^+^ macrophages exhibited a reduced expression of lipoprotein clearance-related genes, such as *ABCA1* and *ABCG1*. Moreover, arteries from PLWH had increased triglycerides and decreased levels of retinoic acid. Finally, in a large biobank, we also observed that genetically regulated gene expression of *ABCA1* and *CXCL12* was significantly associated with atherosclerotic phenotypes in individuals of European and African ancestry, and these associations were partly influenced by individuals with HIV diagnosis, indicating that genetic variations that influence the expression of inflammatory genes may be linked to clinical diagnosis of atherosclerotic phenotypes.

The positive association between HIV copy number and the percent of CD8^+^ cells and CD68^+^ cells within coronary arteries suggests a role for HIV persistence with coronary artery inflammation and is consistent with previous studies.^40^ A study by Hsue et al. found that PLWH have higher levels of inflammatory markers and endothelial dysfunction, which were associated with an increased risk of CVD in PLWH.^4^ Digital spatial profiling of immune-related proteins in coronary plaques by HIV status revealed differential expression of several proteins. Notably, coronary arteries from PLWH had higher expression of several proteins, including STING, CD163, VISTA, and CTLA-4, which are important in immune activation, inflammation, and apoptosis.^25,41–43^ These findings suggest that immune-related proteins may play a role in the pathogenesis of atherosclerosis in PLWH.

Higher expression of *CD163* RNA transcripts in medium-sized coronary arteries from PLWH is consistent with previous research by Burdo et al., which found elevated levels of soluble CD163 (sCD163) in PLWH and a significant correlation between sCD163 and increased noncalcified plaque burden. Notably, this correlation remained significant even after accounting for traditional risk factors.^23^ Other studies have demonstrated that sCD163 is elevated in the peripheral blood of PLWH.^44,45^ Moreover, increased levels of plasma sCD163 have been associated with the activation of T-cells and the expansion of less differentiated effector CD8^+^ T cells, which can lead to poor clinical outcomes in PLWH.^46^ Additionally, elevated levels of sCD163 have been identified as a predictor of all-cause mortality in PLWH.^47^ This may be partly due to increased HIV infection in CD163^+^ macrophages.^48^ Beyond HIV, CD163^+^ macrophages are also associated with high HIF1a expression and plaque progression due to increased angiogenesis and vulnerable plaque.^25,49,50^ Additionally, CD163^+^ macrophages can induce endothelial-to-mesenchymal transition within the fibrous cap.^26^

Increased plasma CXCL12 levels have previously been associated with an increased risk of CAD in the general population.^51^ Despite the systemic increase in CXCL12, local production of CXCL12, such as by arterial endothelial cells, is thought to be pro-atherogenic.^52^ In our study, we observed higher levels of CXCL12 expression in the atheroma of PLWH compared to PWoH, predicted to be expressed by fibroblasts. However, the role of the CXCL12-CXCR4-ACKR3 axis is complex. This is because both CXCR4 and ACKR3 bind other ligands and have also been shown to promote plaque regression and stability.^53^ In summary, therapeutically targeting this axis is complex because of the dual and opposing roles of its components.

The decreased levels of retinoic acid observed in aorta samples from PLWH could be due to multiple reasons, including a previously published mechanism in which the ABCA1 receptor is blocked in the gut by HIV, reducing the absorption of retinoic acid.^54^ Low retinoic acid and cholesterol in cells are associated with an increased ability of HIV to replicate.^55^ Retinoic acid has also been found to have potential benefits in preventing CVD.^56^ Some studies have shown that retinoic acid can help reduce inflammation, oxidative stress, and plaque buildup in the arteries, all of which are risk factors for CVD. Additionally, retinoic acid has been shown to improve lipid metabolism and reduce the risk of atherosclerosis.^57^ Therefore, addressing metabolic and lipid imbalances such as low retinoic acid and high triglyceride levels could be a potential therapeutic strategy for reducing the risk of CVD in PLWH.

This study has shown that we can obtain significant information to understand mechanisms important in the pathogenesis of cardiovascular disease using coronary arteries and aorta from human donors. With newer techniques, we can overcome the barriers of poor RNA quality in tissues with high levels of necrosis. The study has some limitations that should be considered. First, while our untargeted metabolomics/lipidomic approach had a wide range of coverage on lipid species, the levels of these species were only semi-quantified and needed more absolute values. Therefore, targeted methods focusing on specific lipid species of interest are necessary to better understand the underlying biological mechanisms. The absence of longitudinal data hampers our understanding of the time-based dynamics of coronary artery disease in individuals with and without HIV, which might have impacted our results. Despite these limitations, we use multimodal data to identify spatially restricted, HIV-specific inflammatory mechanisms that drive CVD. This approach can provide new insights into the pathogenesis of atherosclerosis in PLWH and identify new therapeutic targets.

## ONLINE METHODS AND MATERIALS

### Selection of coronary artery samples

We obtained epicardial coronary arteries of 13 PLWH and 15-HIV PWoH who died of sudden cardiac death from the tissue biobank at CVPath Institute (Gaithersburg, MD) (**Table S1**). CVPath maintains a curated biorepository of coronary artery beds from over 7000 autopsy hearts from the Office of the Chief Medical Examiner of the State of Maryland (OCME-MD) collected between 2005 and 2019. Each heart was examined by a cardiac pathologist for staging, and, where available, the cause of death was documented alongside anonymized demographic details, including age, sex, and race. Each specimen was fixed in 10% formalin, and regions of interest were decalcified before processing. The arteries with coronary plaques were fixed, and serial sections were embedded in paraffin. Sections were cut at 5–6mm and mounted on charged slides^58^. Aorta (FFPE blocks and frozen tissue) were obtained from the National Disease Research Interchange (NDRI). Selection criteria for samples obtained from NDRI included age (21–80 years), with and without HIV (**Table S2**). Exclusion criteria included chemotherapy or radiation use, Diabetes Type I, and autoimmune disease. Our study was approved by the institutional review board at Vanderbilt University Medical Center.

### Droplet digital PCR

We used DNA droplet digital polymerase chain reaction (DNA ddPCR) to detect as little as one single copy of HIV/CMV per one million cells in coronary arteries from PWH. To this end, we used the long terminal repeat (LTR) primer multiplexed with the RNaseP housekeeping gene (RPP30) (**Table S3**). These primers and reactions were set up as previously published.^18^ The positive droplet threshold was determined using the no template controls on the same run. For the CMV ddPCR, we used the following primers: UL44 forward (FOR) 5ʹ- TACAACAGCGTGTCGTGCTCCG-3’, Reverse (REV) 5ʹ- GGCGTGAAAAACATGCGTATCAAC-3’ and probe 5ʹ 6-FAM- CTATACGCAACGTGCACGGCAG-BHQ1 3ʹ. US28 FOR 5ʹ- TTTGGTGGATCTTTGCCGTG-3’, REV 5ʹ- ACGAAAGCACCAAGCATGAGTTC-3’ and probe 5ʹ- 6-FAM- ATCGCCATTCCACACTTTATGGTGGTG-BHQ1–3’. VIL-10 FOR 5ʹ- TGTTGAGGCGGTATCTGGAGA-3’, REV 5ʹ- CCGTCTTGAGTCCGGGATAG-3’ and probe 5ʹ- 6-FAM- TTTCCCGCAGGCGACCACG- BHQ1–3’. We performed RNA droplet digital PCR using the same LTR and RPP30 primers separately using the one-step RT-ddPCR advanced kit for probes from Bio-Rad and followed the kit protocol by the manufacturer. For the RT-ddPCR, 20ul PCR mixture was prepared using 5ul of RT- probes SuperMix, 1ul DTT(300Mm), 2ul RT enzyme; 1ul Primer/probe (900nM and 250Nm), RNA, and water accordingly to make a total volume. The thermocycling conditions were 50°C (60 min), 95°C (10 min), 40 cycles of 95°C (30 s) with 60°C (1 min) (2°C/s ramp rate); final enzyme deactivation 98°C for 10 min; 4°C: until plate read.

### Immunohistochemical staining

Formalin-fixed paraffin-embedded (FFPE) sections were stained using Movat pentachrome, hematoxylin, and eosin (H&E). Immune cells within the plaques were identified through immunohistochemical (IHC) staining with antibodies targeting CD3 (Roche, Cat# 790–4341, Clone 2GV6, pre-diluted), CD4 (Roche, Cat# 790–4423, Clone SP35, pre-diluted), CD8 (Roche Cat # 790–4460, Clone SP56, pre-diluted), CD68 (Roche, Cat# 790–2931, Clone KP-1, pre-diluted), CD163 (Leica, Cat# NCL-L-CD163, Clone !0D6, 1:50 dilution), vascular cell adhesion molecule 1 (VCAM-1) (Abcam, ab134047, Clone EPR5047, 1:500 dilution), and CX3CR1 (Abcam, ab8020, polyclonal, 1:1000 dilution). Detection was carried out using the DISCOVERY OmniMap anti-Ms HRP (Cat# 760–4310) or anti-Rb HRP (Cat# 760–4311) systems, followed by development with the NovaRed kit (Vector Laboratories). Imaging was performed using an Axio Scan.Z1 (Zeiss, Germany) with a 20X objective. As reported in prior studies, IHC staining was quantified in the segments showing the most severe stenosis using the area quantification module in the HALO image analysis platform (Indica Labs, Corrales, NM).^25,58^

### Fluorescence Labeling of the aorta with HIV p24, anti-CD3 and CD14

5μm FFPE sections were deparaffinized, followed by antigen retrieval. The sections were washed in PBS for 5 minutes and treated with 100 mM glycine for 15 minutes to quench autofluorescence. The sections were incubated for 30 minutes in a blocking buffer of 5% NGS (Normal Goat Serum) in 0.1M PBS to block non-specific binding sites. The primary antibodies were mouse anti-HIV p24 (Fisher Scientific, Cat# 50–220-4167, clone ABM019, 1:100) and rabbit anti-CD3 (Roche Diagnostics, Cat# 790–4341, clone 2GV6, 1:100) in 0.1M PBS or 1% NGS. These were incubated overnight at 4°C. The following day, the sections were washed in PBS for 3x5 minutes and then incubated with secondary antibodies, goat anti-mouse Alexa 568 and goat anti-rabbit Alexa 488 (both at 1:200 dilution in 0.1M PBS), for 120 minutes at room temperature. After incubation with the secondary antibodies, the sections were washed in PBS for 3x5 minutes and fixed in 4% PFA for 15 minutes, followed by another PBS wash for 15 minutes. The sections were then re-blocked with 5% NGS in 0.1M PBS for 30 minutes before applying rabbit anti-CD14 (Invitrogen, Cat# PA5–29588, polyclonal, 1:100 in 1% NGS) and incubated overnight at 4°C. Following this, the sections were washed in PBS for 3x5 minutes and incubated with the secondary antibody, goat anti-rabbit Alexa 647 (1:200 in 0.1M PBS), for 120 minutes at room temperature. The sections were washed in PBS three times for five minutes and followed by incubation with a secondary antibody, goat anti-rabbit Alexa 647 (1:200 in 0.1M PBS), for 120 minutes at room temperature. The sections were then rewashed in PBS for 3x5 minutes. Finally, the sections were mounted with Vector Shield mounting medium containing DAPI for nuclear staining.

### iTEM of aorta sections with HIV p24, anti-CD3 and CD14

Initially, FFPE sections were deparaffinized, followed by antigen retrieval. After PBS washing for 5 minutes and treatment with 100mM glycine for 15 minutes to quench autofluorescence, the sections were blocked for 30 minutes with a locking buffer (5% bovine serum albumin (BSA), 5% NGS, and 0.2% Cold Water Fish Skin Gelatin (CWFS)). Primary antibodies, including mouse anti-HIV p24 (Abbomax, Fisher Catalog# 50–220-4167, ABM019, 1:50) and rabbit anti-CD3 (Roche Diagnostics Confirm Anti-CD3 (Roche, Catalog# NC0402763, Clone 2GV6), 1:100), were added in 0.1M PBS or 1% NGS and incubated overnight at 4°C. The next day, the sections were washed in incubation buffer for 3x5 minutes and then incubated overnight with gold-conjugated secondary antibodies, goat anti-mouse 6nm (Jacksonimmno, Cat# 115–195-146, RRID AB_2338728, polyclonal, 1:20–40) and goat anti-rabbit 12nm (Jacksonimmno, Cat# 111–205-144, RRID AB_2338016, polyclonal, 1:20–40), at 4°C. Following this, the sections were washed in PBS for 6x2 minutes, fixed in 4% PFA for 15 minutes, and washed again in 0.1M PBS for 3x5 minutes. The sections were re-blocked with the same blocking buffer for 30 minutes before incubating overnight at 4°C with rabbit anti-CD14 (Invitrogen, 1:100). After further washing in incubation buffer for 6x2 minutes, the sections were incubated with goat anti-rabbit 18nm gold (Jacksonimmuno, Cat# 111–215-144, RRID AB_2338017, polyclonal, 1:10) for 4 hours. Subsequent PBS washes (6x2 minutes), a 2.5% GAT fixation for 30 minutes, and final washes in 0.1M PBS (3x5 minutes) were performed. The sections were then treated with 1% OsO4 for 30 minutes, washed in 0.1M PBS (3x5 minutes), and stained with 1% uranyl acetate in 50% ethanol for 15 minutes. Finally, the sections were dehydrated through a graded ethanol series (75%, 95%, 100%) and embedded in Epon 12.

### HIV-1 infectivity in PBMCs and THP1 cells

Isolated PBMCs were cultured in RPMI 1640 medium with 10% fetal bovine serum (FBS) and 1% penicillin-streptomycin (R10). Approximately 24 hours before infection, PBMCs (<95 % viability) were treated with human recombinant interleukin-2 (IL-2) at a concentration of 100 IU/mL to stimulate cell proliferation and activation. The PNL4.3 VSVg Luc HIV-1 virus was prepared and titrated to ensure an appropriate infectious dose. PBMCs were seeded in 96-well plates at a density of ~1×10^6^ cells per well. Cells were infected with the PNL4.3 VSVg Luc HIV-1. After adding the virus, cells were centrifuged at 500 rpm for 1 hour to allow virus adsorption. Low-density lipoprotein (LDL), very low-density lipoprotein (VLDL), and high-density lipoprotein (HDL) were prepared and quantified. Each lipoprotein was added to the respective wells at a final concentration of 20 μg/mL, where control wells received no lipoprotein treatment. After adding lipoproteins, cells were incubated for 48 hours at 37°C in a 5% CO2 atmosphere. THP1 cells were infected, as above.

### Luciferase Assay for HIV-1 infectivity

After the infection step and 48 hours of incubation with lipoproteins and controls, we removed the culture medium and lysed the cells using the Promega Luciferase Cell Culture Lysis Buffer (Promega Glo Lysis Buffer 1X). The lysates were transferred to a black 96-well assay plate. Luciferase activity was measured using the Promega Luciferase Assay System according to the manufacturer’s instructions. Luminescence was quantified using a luminometer. Relative light units (RLU) were recorded for each well. The infectivity was expressed as RLU, normalized to the control wells without lipoprotein treatment. Results were expressed as triplicate experiments with the mean ± standard deviation (SD).

### Clonal hematopoiesis of indeterminate potential (CHIP)

We identified CHIP mutations in DNA extracted from the coronary arteries using hybrid capture oligonucleotide probes. We extracted DNA from serial sections of FFPE coronary arteries, followed by barcoding and library preparation, as published.^59^ The libraries were pooled, and hybrid capture technology was used to selectively enrich CHIP regions from the pooled DNA libraries. Sequencing was performed on an Illumina NovaSeq 6000. Putative somatic mutations were identified using the Mutect2 version 4.1.0.0–GATK version 4.1.4.1 software package. Filtering criteria included the removal of variants with low total read depth (<100), low variant allele read depth (<3), and a variant allele frequency (VAF) below 2%.^59^

### Digital spatial analysis - Nanostring

Expression of multiple immune-related proteins was measured on 5μm thickness formalin-fixed paraffin-embedded (FFPE) tissue sections from the coronary plaques of two male HIV-positive and HIV-negative with the highest degree of immune cell infiltration. FFPE sections were treated 104 with citrate buffer (pH6) for antigen retrieval. They were stained with fluorescent anti-CD3 (Origene, Cat# UM500048, clone UMAB54), anti-CD8 (Invitrogen, Cat# 14–0008-82, clone AMC908), anti- CD68 (Satna Cruz, Cat.# sc-20060, clone KP1) and SYTO 83 nuclear staining (Invitrogen, Cat# S11364). Fluorescence microscopy on the GeoMX^®^ platform was used to image the slides. Twelve regions of interest/areas of interest (ROIs/AOIs) from each were processed using the NanoString’s GeoMX^®^ digital spatial profiling platform (https://www.nanostring.com/scientific-content/technology-overview/digital-spatial-profiling-technology). Images of stained sections were captured at 20X magnification. ROIs/AOIs for molecular profiling were selected as geometric shapes in macrophage-abundant, T-cell-abundant, and adipose tissue sections. For the protein profiling assay, the FFPE slides were bathed in a multiplexed cocktail of primary antibodies with photocleavable DNA-indexing oligos (GeoMX^®^ Immune profile core (Nanostring, GeoMx Imm Cell Pro_Hs, Item 121300101), Immune Cell typing (GeoMx Imm Cell Typing_Hs, Item 121300104), Immune Activation Status (GeoMx Imm ActivateStatus_Hs, item 121300103), IO Drug Target (GeoMx IO Drug Target_Hs, 121300102), cell death (GeoMx Cell Death Mo_Hs_nC, 121300112), and Pan Tumor modules (GeoMx Pan-Tumor_Hs, item 121300105). AOIs were exposed to ultraviolet (365nm) to release the indexed oligos/barcodes for collection. Oligos were captured from the AOI by microcapillaries and dispensed into 96-well plates. Following collections from all AOIs, the oligos (hybridized to unique four-color, six-spot optical indexing barcodes) were quantified on the nCounter analysis platform. Data were normalized to area; signal-to-noise ratios (SNR) were calculated using isotype controls. Proteins with SNR less than two were not included in differential expression analysis. Data were visualized by unsupervised hierarchical clustering. Differential gene expression was analyzed by unpaired t-test with Benjamini Hochberg (BH) correction. For the RNA analysis, we the FFPE sections were stained with the morphology kit (GeoMx Solid Tumor Morp Kit HsR, cat# 121300310), and the CD45+ areas were used to select regions of interest for analysis. We performed a targeted analysis using the GeoMX cancer transcriptomic atlas (GeoMx RNA Slide Prep FFPE PCLN, item# 121300313, Master Kit--12 reactions, item#100052, GeoMx NGS RNA CTA Hs, item 121400101, GeoMx Hyb Code Pack_RNA, item#121300402, and GeoMx Seq Code Pack_AB, item #121400201). For the Whole Transcriptomic Analysis, we used the GeoMX Morphology kit to define regions of interest to select areas with CD45+ cells (GeoMx NGS RNA WTA Hs, item# 121401102, GeoMx RNA Slide Prep FFPE PCLN, item# 121300313, GeoMx Seq Code Pack_AB, item# 121400201). Like the protein assay, AOIs were exposed to ultraviolet (365nm) to release the indexed oligos/barcodes for collection, and the genes in each section were quantified by sequencing.

### Visium

The manufacturer’s recommendations for tissue mounting on the Visium Spatial Gene Expression slide (10x Genomics, Pleasanton, CA, USA, 1000338) were followed. Each tissue section was placed within one of four 6.5 × 6.5 mm capture regions of the Visium slide. Each of the capture areas contains an array of ~5000 spots (55 μm in diameter), which have oligonucleotide sequences required for the capture of probe-ligated mRNA, a spatial barcode, unique molecular identifier (UMI), and Illumina sequencing primer binding site.

Sections were left overnight to dry and incubated the following day in a 60-degree fan-forced oven for 2 hours and stained with H&E. H&E-stained slides were coated with 85% glycerol and a coverslip and imaged with the Olympus VS120 Slide Scanner at 40× lens magnification. After imaging, glycerol was washed off by immersing the Visium slide into the beaker containing 800 mL of Milli-Q water. The slide was immersed horizontally and held until the coverslip fully separated. Sections were dried at 37 °C and stored at 4 °C before decrosslinking. Samples were decrosslinked using the recommended TE buffer (pH 9.0) before human transcriptome probes covering 18,000 genes were ligated to target mRNA for capture and library construction according to the Visium Spatial Gene Expression for the FFPE protocol with no deviation. The optimal PCR cycle number determination was performed using the 10X recommended protocol for qPCR on the QuantStudio 12K instrument for PCR indexing.

The final library quality was assessed using the Agilent Bioanalyzer and quantified using a qPCR-based method with the KAPA Library Quantification Kit (P/N: KK4873) and the QuantStudio 12K instrument. Prepared library pools were pooled in equimolar ratios, and the resulting pool was subjected to cluster generation using the NovaSeq 6000 System, following the manufacturer’s protocols. 150 bp paired-end sequencing was performed on the NovaSeq 6000 platform, targeting 250M reads per sample. Raw sequencing data (FASTQ files) obtained from the NovaSeq 6000 was subjected to quality control analysis, including read quality assessment. Real-Time Analysis Software (RTA) and NovaSeq Control Software (NCS) (1.8.0; Illumina) were used for base calling. MultiQC (v1.7; Illumina) was used for data quality assessments.

This 1000-gene panel included 243 genes for cell typing and mapping, 269 genes for cell state and function, 435 for cell-cell interaction (ligand-receptor), and 46 for hormone activities. The eight coronary arteries included four early and four late atheroma. We used the Nanostring morphology markers to select the fields of view: Pan-CK and DAPI, CD45 (1GeoMx Solid Tumor Morp Kit HsP, cat# 121300310). For CoSMX, we used machine learning-based cell segmentation to allow for single-cell resolution of spatial data analyzed as previously published.^60^

### CosMx Spatial Molecular Imager (SMI)

#### Sample preparation:

We took Formalin-Fixed Paraffin-Embedded (FFPE) sections of coronary arteries from People Living With HIV (PLWH, n=4) and People Without HIV (PWoH, n=4) who had coronary artery disease. These sections were placed on VWR^®^ Superfrost^®^ Plus slides and left to bake overnight at 60°C. This step was crucial in ensuring the tissue adhered to the glass slides. To deparaffinize the tissue, we washed it twice for 5 minutes in Xylene and twice for 2 minutes in 100% Ethanol. Following this, we subjected the tissue to proteinase K digestion (3 μg/ml; ThermoFisher, 40°C for 30 min), and heat-induced epitope retrieval (HEIR, 100°C for 15 min in Leica buffer ER1 conditions) to make the target RNAs and epitopes accessible. The coronary arteries were then rinsed twice with diethylpyrocarbonate-treated water (DEPC H2O). After this, we incubated them with 1:1000 diluted fiducials (Bangs Laboratory) in 2X saline sodium citrate, 0.001% Tween 20 solution for 5 minutes at room temperature. Any remaining fiducials were removed by rinsing the samples with 1X phosphate-buffered saline (PBS). Subsequently, we fixed the samples with 10% neutral buffered formalin (NBF) for 5 minutes at room temperature. After fixing, we rinsed the coronary arteries with Tris-glycine buffer (0.1M glycine, 0.1M Tris-base in DEPC H2O) and 1X PBS for 5 minutes each. We then blocked the samples using 100 mM N-succinimidyl acetate (NHS-acetate, ThermoFisher) in NHS-acetate buffer (0.1M NaP, 0.1% Tween pH 8 in DEPC H2O) for 15 minutes at room temperature. Finally, the samples were rinsed with 2X saline sodium citrate (SSC) for 5 minutes, and an Adhesive SecureSeal Hybridization Chamber (Grace Bio-Labs) was attached to cover them. Then, coronary arteries were incubated with a fluorophore-conjugated antibody cocktail against CD298/B2M (Nanostring master mix, CD298 EP1845Y, B2M D8P1H, Cat# 121500120), CD45/PanCK master mix (CD45 D9M8I, PanCK AE-1/AE-3, Cat# 121500121), CD68 (Satna Cruz, Cat.# sc-20060, clone KP1), and DAPI (Cat# 121303304) at room temperature for 1 hr. After antibody morphology staining, the samples were washed twice using 2X SSC for 2 min at room temperature. Custom-made slide covers were attached to the sample slide to form a flow cell.

#### Spatial transcriptomic measurement using CosMx SMI:

The SMI instrument was used for target RNA reading, per standard procedures.^61^ Briefly, the prepared flow cell was placed onto the SMI device, and the specimens were cleansed with Reporter wash buffer to purge air bubbles. Upon placement of the flow cell onto the machine, the whole flow cell was scanned, and approximately 20 fields of view (FOVs) were positioned on each slide for RNA reading. The initial cycle of RNA reading commenced with the introduction of 100 μL of Reporter Pool 1 into the flow cell, allowing for a 15-minute incubation period. After incubation, 1 mL of Reporter Wash Buffer was run across the flow cell to eliminate the Reporter probes that were not bound, which was succeeded by substituting Reporter Wash buffer with Imaging buffer before the imaging process. Nine Z-stack images (0.8 um step size) of each FOV were captured, followed by the UV cleavage and wash-off of fluorophores on the reporter probes using Strip Wash buffer. This fluidic and imaging procedure was repeated for the remaining 15 reporter pools, and the 16 cycles of reporter hybridization-imaging were conducted eight times to enhance RNA detection sensitivity. After nine complete RNA reading cycles, nine Z-stack images for 5 channels (4 antibodies and DAPI) were captured after unbound antibodies and DAPI were rinsed with Reporter washing buffer, and the flow cell was filled with Imaging buffer.

#### Image processing and feature extraction:

An internal SMI data processing pipeline^61^ was utilized for raw image processing and feature extraction, including registration, feature detection, and localization. 3D rigid image registration was executed using fiducials embedded in the samples, aligned with the fixed image reference created at the start of the SMI run to correct any shift. An RNA image analysis algorithm was then used to pinpoint reporter signature locations in the X, Y, and Z axes, along with the assigned confidence. These reporter signature locations and associated features were compiled into a single list. The XYZ location information of individual target transcripts was then extracted and documented in a table using a secondary analysis algorithm, as outlined.^61^

#### Cell segmentation:

The Z-stack images of immunostaining + DAPI were utilized to outline cell boundaries on the samples. A cell segmentation pipeline using a machine learning algorithm^62,63^ was employed to map transcripts accurately to cell locations and subcellular compartments. The transcript profile of individual cells was created by merging the target transcript location and cell segmentation boundaries. Cells with less than 20 total transcripts assigned were excluded from the analysis.

#### Cell type annotation:

Cell type annotation was conducted by comparing expression observed in each cell to expression profiles of cell types using a negative binomial model within the InSituType package (https://github.com/Nanostring-Biostats/InSituType). The reference cell profiles were sourced from Danaher et al, 2022.^64^ Cell assignments were confirmed by comparing to known marker gene expression, protein expression, and location within tissue spatial organization. *Spatial neighborhood / Niche annotation*: The cell type composition of cells within a 50um radius in 2D physical space of each cell was calculated and grouped into 7 clusters using the MClust R package.^65^ The resulting niches or neighborhood types were identified based on cell type composition and spatial location within the tissue structure.

### Western blot analysis

We used Western blot to investigate CD163, CD209, and CXCL12 expression levels in aorta tissues. β-actin was also included as a loading control. Protein extraction was performed by homogenizing the tissues in RIPA buffer containing protease and phosphatase inhibitors (Sigma-Aldrich), and the protein concentration was determined using the BCA protein assay kit. The protein samples were separated on 10–15% SDS-PAGE gels (Bio-Rad) for CD163 (Abcam, cat# ab87099, polyclonal), CXCL12 (Cell Signaling, cat# 3740S), CD209 (Cell Signaling, cat# 13193S, clone D7F5C), actin (Abcam, cat# ab8227, polyclonal), and GAPDH (Abcam, cat # ab8245, clone 6C5) was separated on a 12% SDS-PAGE gel. The protein samples were loaded in equal amounts (30 μg) onto the gels and separated by electrophoresis. The separated proteins were transferred onto nitrocellulose membranes (Bio-Rad) using the Trans-Blot Turbo Transfer System (Bio-Rad) and blocked with 5% non-fat milk in TBST buffer (Tris-buffered saline with 0.1% Tween-20) for one and half hour at room temperature. The primary antibodies against the target proteins were incubated overnight at 4 °C in 5% BSA in TBST buffer. The membranes were washed with TBST buffer three times for 5 minutes each and then incubated with HRP-conjugated secondary antibody diluted in 5% non-fat milk in TBST buffer for 1 hour at room temperature. The membranes were rewashed with TBST buffer thrice for 10 minutes each and visualized using enhanced chemiluminescence (ECL) substrate. Blots were imaged using the *Li-Cor* machine, which allows for detecting and quantifying chemiluminescent signals from the Western blots.

### Evaluation of genetically regulated gene expression associations in the Vanderbilt University Medical Center biobank, BioVU.

Vanderbilt University Medical Center curates a large biobank, BioVU. This repository includes blood samples collected during routine patient care, which are genotyped and linked to de-identified electronic health records for the patient.^66^ BioVU has collected over 300,000 samples to date.^67^ We leveraged the BioVU dataset to assess whether there were differences in imputed gene expression values among the genes *ABCA1, CD163,* and *CXCL12*, in individuals with diagnoses of atherosclerotic phenotypes: ischemic heart disease, coronary atherosclerosis, and myocardial infarction (phecodes: 411, 411.4, and 411.2, respectively). Individuals were required to have at least two instances of a phecode to be labeled as a case, controls were defined by having none of the respective atherosclerotic phecodes. Phecodes were mapped from ICD9/10 billing codes using the PheWAS package (version 0.99.5–2) in R (version 3.6.0).^68^ Genotype data was collected using Illumina’s Multi-Ethnic Genotyping Array (MEGA), and quality control was performed as previously described.^69,70^ Genetically regulated gene expression (GREX) was calculated using genotype data to estimate the genetically regulated component of gene expression across 49 tissues (i.e. local expression quantitative trait loci derived from the Genotype Tissue Expression Project GTEX version 8 dataset, Table S6).^71^ Three distinct gene expression imputation models were tested, and the model with the best performance (highest prediction performance r^2^) for each gene-tissue pair was preserved for analysis.^72–74^ Logistic regression models stratified by genetic ancestry group (European ancestry n=65,364; African ancestry n=12,314) were performed to test for the association between *ABCA1*, *CD163*, or *CXCL12* GREX (predictor variables) between atherosclerosis cases and controls (outcome variables). The covariates used in these models included current age, median age of medical record, BMI, sex, genotype batch, and principal components 1–10. P-values below the Bonferroni-corrected p-value threshold were considered significant (i.e., 0.05/# of genes*phenotypes tested), while associations with p < 0.05 were considered nominally significant.

### Sensitivity analyses in BioVU individuals with HIV diagnosis

We defined HIV cases in BioVU using ICD9/10 billing codes for HIV (**Table S5**), where individuals were required to have two instances of the HIV codes to be considered a case. Individuals with only a single example of the HIV codes or those with any of the exclusion HIV codes (**Table S5**) were removed from the analysis. Non-HIV controls were defined as individuals without any of the HIV case inclusion codes or HIV case exclusion codes in their medical records. We identified 1,361 HIV cases and 58,806 HIV controls, which were used for the sensitivity analyses, and further subset into individuals of European genetic ancestry (n= 707 cases/49,716 controls) or African genetic ancestry (n= 654 cases/9,090 controls). We performed logistic regression analyses to assess the association between the GREX of *ABCA1, CD163*, or *CXCL12* (predictor variables) with the atherosclerosis phenotypes (outcome variables) described above. For these sensitivity analyses, we used the following covariates: current age, the median age of the medical record, BMI, sex, genotype batch, principal components 1–10, and HIV diagnosis.

### Metabolomics

Frozen aorta tissues were weighed and ground with liquid nitrogen using a cryomill (Retsch) at 25 Hz for 45 seconds. Tissues were then extracted using a solvent consisting of 40:40:20 acetonitrile: methanol: water +0.5% FA +15% NH4HCO3^75^ at a volume of 40 μL per 1 mg of tissue. We incubated the samples on dry ice for 10 minutes, followed by centrifugation for 30 minutes at 16,000 g. We carefully transferred the resulting supernatants to new Eppendorf tubes and performed a second centrifugation at 16,000 g for 25 minutes to remove residual debris before LC-MS analysis.

The extracts were examined within 24 hours using liquid chromatography paired with mass spectrometry (LC-MS). The LC-MS technique was based on hydrophilic interaction chromatography (HILIC) coupled to the Orbitrap Exploris 240 mass spectrometer (Thermo Scientific).^76^ The LC separation was performed on a XBridge BEH Amide column (2.1 × 150 mm, 3.5 *μ*m particle size, Waters, Milford, MA). Solvent A is 95%: 5% H2O: acetonitrile with 20 mM ammonium acetate and 20mM ammonium hydroxide, and solvent B is 90%: 10% acetonitrile: H2O with 20 mM ammonium acetate and 20mM ammonium hydroxide. The gradient was 0 min, 90% B; 2 min, 90% B; 3 min, 75% B; 5 min, 75% B; 6 min, 75% B; 7 min, 75% B; 8 min, 70% B; 9 min, 70% B; 10 min, 50% B; 12 min, 50% B; 13 min, 25% B; 14min, 25% B; 16 min, 0% B; 18 min, 0% B; 20 min, 0% B; 21 min, 90% B; 25 min, 90% B.^77^ The following parameters were maintained during the LC analysis: flow rate 150 mL/min, column temperature 25 °C, injection volume 5 μL, and autosampler temperature 5 °C. The mass spectrometer was operated in positive and negative ion mode to detect metabolites. The following parameters were maintained during the MS analysis: resolution of 180,000 at m/z 200, automatic gain control (AGC) target at 3e6, maximum injection time of 30 ms and scan range of m/z 70–1000. Raw LC/MS data were converted to mzXML format using the command line “msconvert” utility.^78^ Data were analyzed via the EL-MAVEN software version 12.

### Lipidomics

#### Tissue homogenization and extraction for lipids:

Aorta tissues were homogenized using a Retsch CryoMill. We mixed the homogenate with 1 mL of Extraction Buffer containing IPA/H2O/Ethyl Acetate (30:10:60, v/v/v) and Avanti Lipidomix Internal Standard (diluted 1:1000) (Avanti Polar Lipids, Inc. Alabaster, AL). Samples were vortexed and transferred to bead mill tubes for homogenization using a VWR Bead Mill at 6000 g for 30 seconds, repeated twice. The samples were sonicated for 5 minutes and then centrifuged for 5 minutes at 15,000 g/4°C. The upper phase was transferred to a new tube and kept at 4°C. Another 1 mL of Extraction Buffer (30:10:60, v/v/v) was added to the tissue pellet-containing tube to re-extract the tissues. The samples were vortexed, homogenized, sonicated, and centrifuged. The supernatants from both extractions were combined, and the organic phase was dried under liquid nitrogen gas.

#### Sample reconstitution for lipids:

The dried aorta samples were reconstituted in 300 μL of Solvent A (IPA/ACN/H2O, 45:35:20, v/v/v). After brief vortexing, the samples were sonicated for 7 minutes and centrifuged at 15,000 g for 10 minutes at 4°C. The supernatants were transferred to clean tubes and centrifuged again for 5 minutes at 15,000 g at 4°C to remove any remaining particulates. For LC-MS lipidomic analysis, 60 μL of the sample extracts were transferred to mass spectrometry vials.

#### LC-MS analysis for lipids:

Sample analysis was performed within 24 hours after extraction using a Vanquish UHPLC system coupled with an Orbitrap Exploris 240^™^ mass spectrometer equipped with a H-ESI^™^ ion source (all Thermo Fisher Scientific). A Waters (Milford, MA) CSH C18 column (1.0 × 150 mm × 1.7 μm particle size) was used. Solvent A consisted of ACN:H2O (60:40; v/v) with 10 mM Ammonium formate and 0.1% formic acid, while solvent B contained IPA:ACN (95:5; v/v) with 10 mM Ammonium formate and 0.1% formic acid. The mobile phase flow rate was set at 0.11 mL/min, and the column temperature was maintained at 65 °C. The gradient for solvent B was as follows: 0 min 15% (B), 0–2 min 30% (B), 2–2.5 min 48% (B), 2.5–11 min 82% (B), 11–11.01 min 99% (B), 11.01–12.95 min 99% (B), 12.95–13 min 15% (B), and 13–15 min 15% (B). Ion source spray voltages were set at 4,000 V and 3,000 V in positive and negative mode, respectively. Full scan mass spectrometry was conducted with a scan range from 200 to 1000 m/z, and AcquireX mode was utilized with a stepped collision energy of 30% with a 5% spread for fragment ion MS/MS scan.

Lipidomic data analysis was performed as described.^79^ After normalization, all data were analyzed in R using the *lipidr* package.^80^ All code to analyze data and generate figures can be found at https://github.com/hubertdl/Human_Aorta_HIV_Lipidomics. Data were log-transformed, and only the sets of readings with the highest values were used for lipids, which had multiple readings across replicates. Lastly, lipid names were modified to be compatible with *lipidr*, as described previously (*note*: the code to convert lipid names is available through the GitHub link above).

The lipid composition of aorta samples from PLWH and HIV-negative patients were compared after processing using the “de_analysis” function from *lipidr* with default settings. Here, *lipidr* uses moderated *t*-tests to identify significant differences in lipids between sample type. Significantly different lipids were those with adjusted *p*-values < 0.05 (*note*: *p*-values were adjusted to correct for multiple comparisons using a false discovery rate procedure) and log fold change greater than 1 or less than −1. These results were used to perform a lipid set enrichment analysis using the “lsea” function in which entries were ranked by fold change; only classes with at least four associated lipids were considered, and 100,000 permutations were run. Here, the method *lipdr* used is based on the commonly used gene set enrichment analysis approach previously outlined.^81^ Briefly, lipid class and chain length categories were determined from annotations extracted from lipid names in the data set, and lipids were ranked by fold change. A permutation algorithm was used to calculate enrichment scores and *p*-values for each lipid set. Sets with adjusted *p*-values < 0.05 were defined as significantly enriched. Lastly, heatmaps were generated for significantly enriched lipid classes using the “plot_chain_distribution” function in lipidr.

### Other statistical analysis

The individual sections include details of statistical analysis of the metabolomic and lipidomic data. Differential protein expression in coronary plaques and correlation plots of protein expression were analyzed using a t-test on the GeoMx software platform with Benjamini Hochberg correction for multiple comparisons, where applicable. Statistical differences in immune cells within coronary plaques of HIV-positive and HIV-negative persons were calculated using GraphPad Prism 10.1.0 and R v.3.6.1.

## Data Availability

All code to analyze the lipidomics and create figures can be found at https://github.com/hubertdl/Human_Aorta_HIV_Lipidomics. All other datasets generated in this study are available from the corresponding author upon request.
